# A TTC19 mutation associated with progressive movement disorders and peripheral neuropathy: Case report and systematic review

**DOI:** 10.1111/cns.14425

**Published:** 2023-11-06

**Authors:** Xianjun Xuan, Jie Ruan, Chunhong Wu, Yiyi Gao, Lingfei Li, Xiaoguang Lei

**Affiliations:** ^1^ Department of Neurology Hangzhou Ninth People's Hospital Hangzhou China; ^2^ Zhejiang Provincial Key Laboratory for Drug Evaluation and Clinical Research, Department of Clinical Pharmacy, the First Affiliated Hospital Zhejiang University School of Medicine Hangzhou China; ^3^ Ningbo Medical Center Lihuili Hospital Ningbo China; ^4^ Department of Neurology, Affiliated Hangzhou First People's Hospital Zhejiang University School of Medicine Hangzhou China; ^5^ First Department of Neurology, First Affiliated Hospital of Kunming Medical University Kunming Medical University Kunming China

## Abstract

**Background:**

Mitochondrial complex III (CIII) deficiency is an autosomal recessive disease characterized by symptoms such as ataxia, cognitive dysfunction, and spastic paraplegia. Multiple genes are associated with complex III defects. Among them, the mutation of TTC19 is a rare subtype.

**Methods:**

We screened a Chinese boy with weakness of limbs and his non‐consanguineous parents by whole exome sequencing and targeted sequencing.

**Results:**

We report a Chinese boy diagnosed with mitochondrial complex III defect type 2 carrying a homozygous variant (c.719‐732del, p.Leu240Serfs*17) of the TTC19 gene. According to the genotype analysis of his family members, this is an autosomal recessive inheritance. We provide his clinical manifestation.

**Conclusions:**

A new type of TTC19 mutation (c.719‐732del, p.Leu240Serfs*17) was found, which enriched the TTC19 gene mutation spectrum and provided new data for elucidating the pathogenesis of CIII‐deficient diseases.

## 1 INTRODUCTION

The mitochondrial respiratory chain (MRC) is composed of five enzyme complexes embedded in the mitochondrial inner membrane, and complex III (CIII) is one of them.^1^ CIII is responsible for transferring electrons from the reduced coenzyme Q to cytochrome C, contributing to the formation of electrochemical potential and adenosine triphosphate.^2^ CIII consists of 11 subunits, of which cytochrome *b* (cyt *b*) is encoded by mitochondrial DNA (mtDNA) and the remaining subunits are encoded by nuclear genes.^3^ CIII defects are associated with variations in assembly factors. The mutations in TTC19 are fairly rare, while the most common mutation is in BCSL1. The TTC19 mutation was first described in four Italian patients from three unrelated kindred, presenting with severe neurodegenerative disease in adults and children.^2^ Here, we report a 13‐year‐old Chinese boy with basal ganglia involvement, manifested with progressive movement disorders, limb weakness, suspicious ataxia, and peripheral neuropathy, with a novel frameshift mutation in the TTC19 gene.

## 2 CASE DESCRIPTION

The patient is a 13‐year‐old boy from Xishuang Banna, Yunnan, China, the first child of non‐consanguineous, healthy parents. The boy had no birth or feeding difficulties. Until the age of 9, the boy felt muscle weakness when jumping, which was fluctuating. At the age of 11, his running posture became weird. The forearms were difficult to bend, and the raising of arms could not be kept for a long time. At the age of 12, the boy had difficulty lifting his right forearm, squatting, and standing up. He got a little choked when eating sometimes. He likes soft drinks, although it was difficult to determine if it was the reason for his eating difficulty. His academic performance in school was obviously poor (about 20%–30% of the total score in common courses and about 30% of the total score in physical courses). He was suspected by a psychiatrist to have attention deficit and hyperactivity disorder, mild mental retardation, and exhibitionism.

**FIGURE 1:**
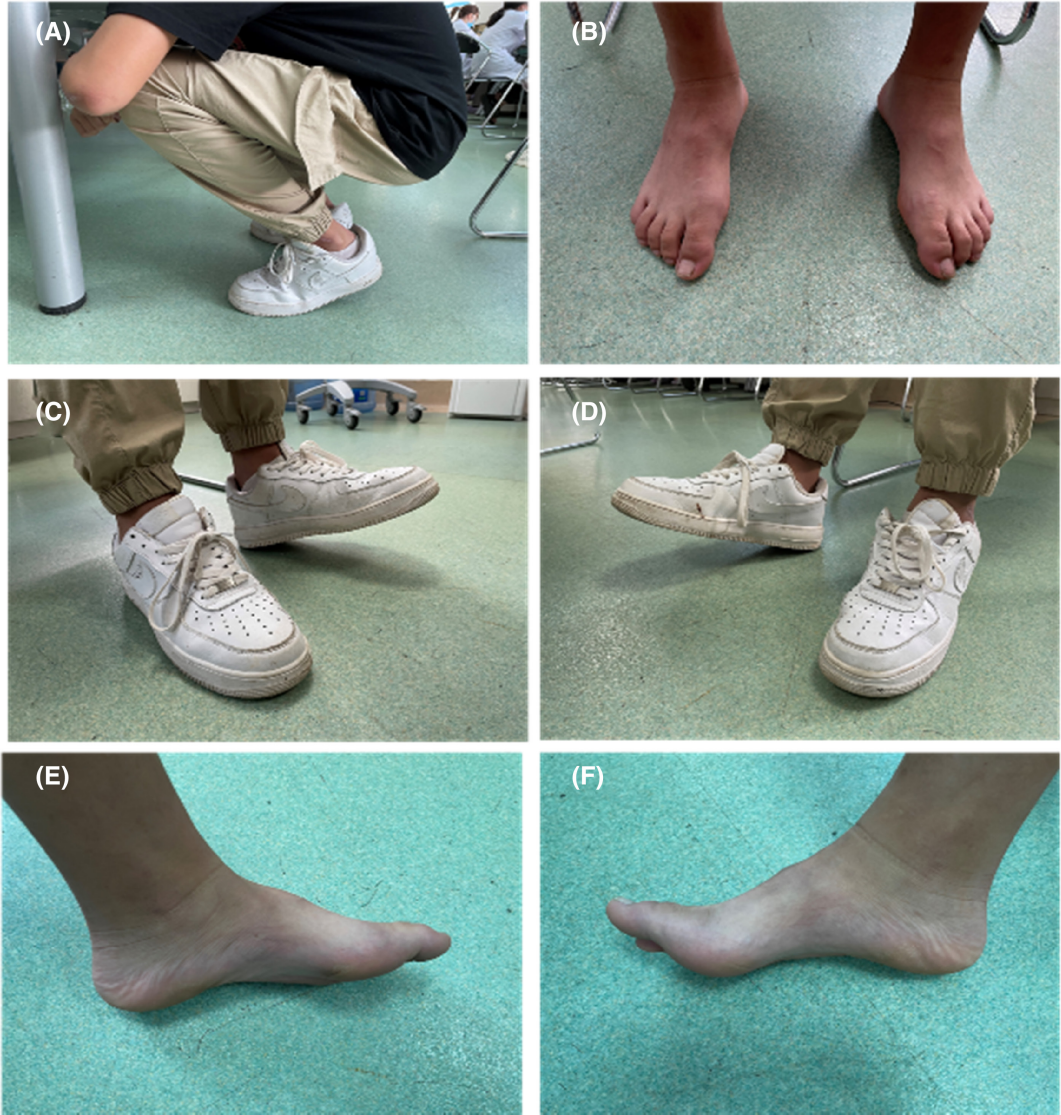
The boy had difficulty in squatting and was unable to land on both his heels when squatting (A); The shape of the boy's feet (B). The boy tried to bend his left foot backwards; The boy tried to bend his right foot backwards (C, D). The foot drop and pes cavus (E, F).

**FIGURE 2:**
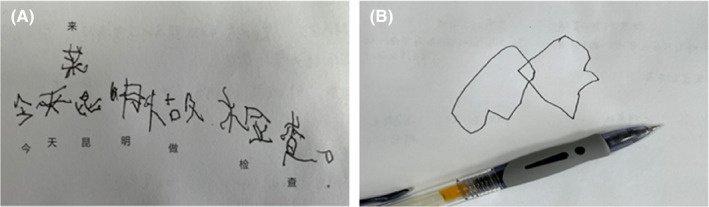
The boy had difficulty in writing (spending about 2.5 min for 8 Chinese characters) and in copying the interlocking pentagons (A, B).

The physical examination showed normal communication without aphasia, mild dysarthria and slowness in speech with normal functions of other cranial nerves, 2/5 errors in the “continuous 100–7” test, normal memory and executive function, muscle strength grade 4 for proximal limbs, no muscle atrophy for distal limbs, difficulty for full squatting, steppage gait with foot drop and pes cavus for both feet (Figure 1), tendon hyperreflexia in bilateral knee reflex and ankle reflex, and negative in pyramidal tract signs. He had moderate unbalance in walking (in steppage gait). When walking, he had mild involuntary movement in the neck, shoulders, and upper limbs (Video S1). He had moderate slowness in performing rapid alternating movements in the right hand (Video S1). He had dystonia and myoclonus‐like movements (for limbs and trunk, but obvious for the right upper limb) when holding arms (Video S1). The tests of coordinate movements (finger‐nose test and heel‐to‐knee test) were impaired partially due to the involuntary movements. He had difficulty in writing (spending about 2.5 min for 8 Chinese characters) and in copying the interlocking pentagons (Video S1 and Figure 2). He felt very itchy when given squeezing to the Achilles tendon and gastrocnemius muscle and had no other obvious sensory problem. The Mini‐mental state examination (MMSE) score was 27/30 (He was in grade 7 in school when taking the test).

**FIGURE 3:**
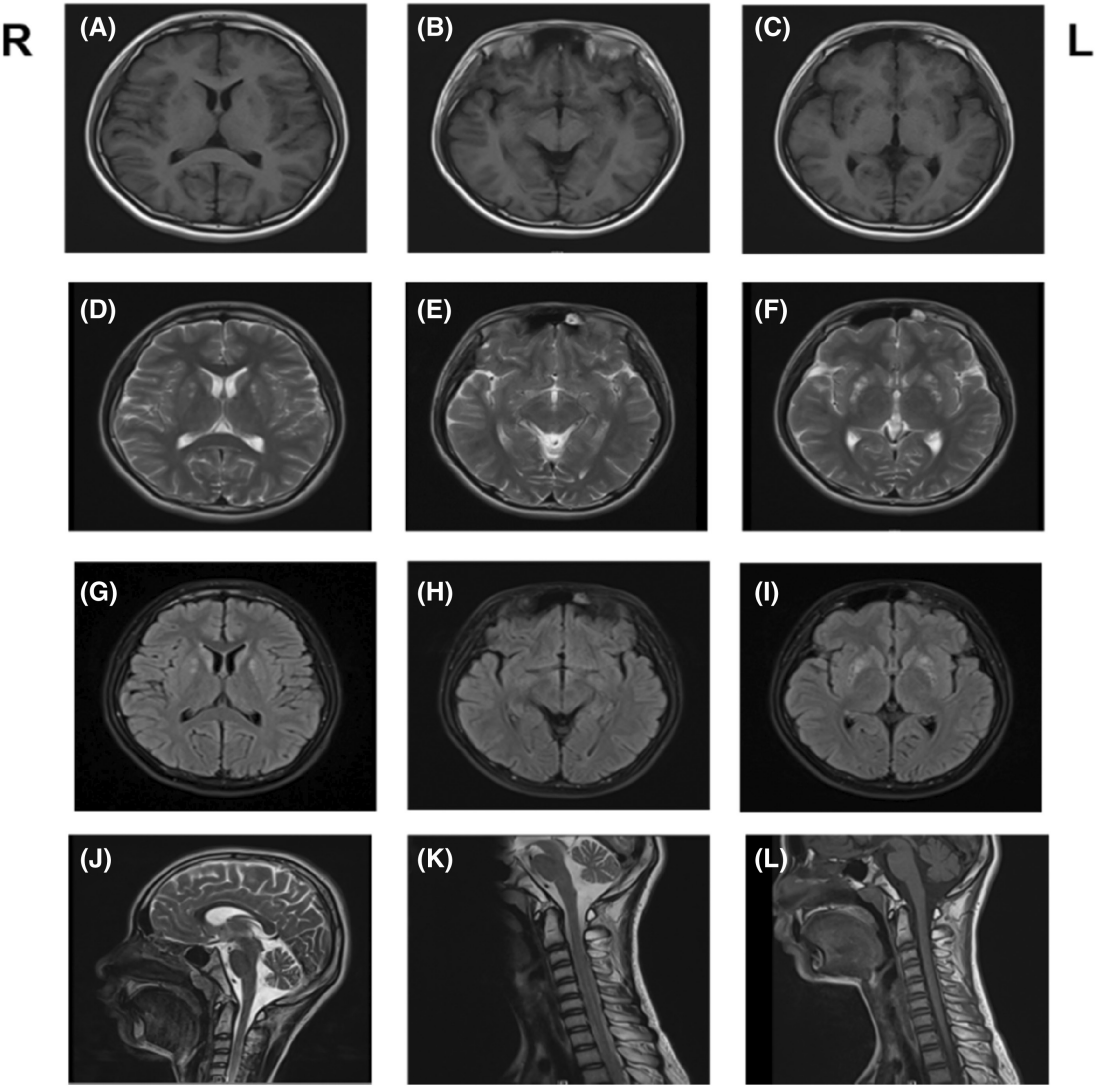
The patient's brain and cervical MRI. Axial T1‐weighted (A‐C), T2‐weighted (D‐F), and Flair (G‐I) MRI shows multiple abnormal signal changes in bilateral basal ganglia. Sagittal T2‐weighted (J) MRI shows a higher signal in the medulla. Sagittal T2‐weighted (K) and T1‐weighted (L) MRI shows abnormal signal changes in cervical cord 2–7.

Laboratory tests showed that ceruloplasmin and thyroid function were normal. Brain MRI showed multiple abnormal signal changes in bilateral basal ganglia and medulla on T1, T2, and fluid‐attenuated inversion recovery (Flair) images. Cervical cord 2–7 showed a slightly higher signal on the T2 image in cervical MRI (Figure 3). Electrophysiology showed chronic neurogenic lesions on the extremities. The motor and sensory fibers of both lower limbs were impaired. The motor fibers were the most affected in the lower limbs, especially neurogenic lesions in the proximal left tibial nerve.

**FIGURE 4:**
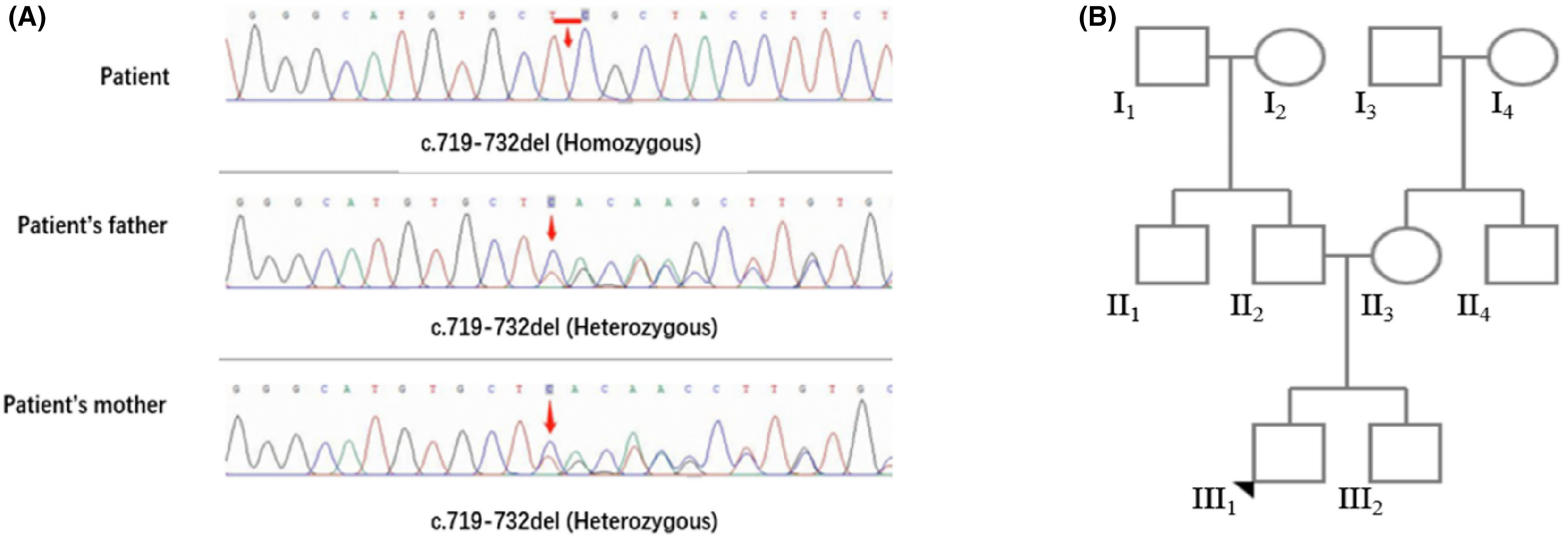
Molecular analysis of TTC19 gene mutation. Mutation analysis of the TTC19 gene. Red arrowhead marks the sites of base alterations. The DNA sequences of the family subjects are shown (A). Pedigree of the family. Square men, circle female, filled symbols affected individual (B).

Whole exome sequencing revealed a homozygous mutation c.719‐732del (p.Leu240Serfs*17) in TTC19. Variation inherited from father and mother is a frameshift mutation in the gene's coding region that can lead to loss of protein function through nonsense‐mediated mRNA degradation or premature termination of the encoded amino acid sequence (Figure 4).

## 3 METHODS

Whole exome sequencing (WES) and Sanger sequencing were performed as in our previous case reports.^4^ Peripheral blood samples were obtained from the boy and his parents to confirm the pathogenic mutation. WES was performed with the IDTXGen Exome Research Panel v1.0 and the Illumina Novaseq 6000 platform, while a whole blood magnetic bead purification kit was applied to extract DNA. Illumina Sequence Control Software (SCS) was applied to analyze the sequencing data. The target mutation was screened and gained in the three genome databases (1000 genomes project_EAS, ExAC, and gnomAD_EAS). Consequently, a variant of TTC19 was found in the boy.

Sanger sequencing, as well as co‐segregation analyses, was performed to identify the mutation site subsequently. The design of primers, the amplification of the fragments using PCR, and the purification of the PCR products were performed step by step (Premier Biosoft, USA; Bioer Technology Co. Ltd., China; Applied Biosystems, Thermo Fisher Scientific). Chromas Lite v2.01 turned out the findings of Sanger sequencing (Technelysium Pty Ltd.).

## 4 DISCUSSION AND CONCLUSION

Mitochondrial disease is a clinical and genetic heterogeneous disease caused by MRC and oxidative phosphorylation dysfunction.^5^ The disease usually affects organs with high energy demands, such as the brain, heart, liver, and skeletal muscle.^6^ The molecular genetic diagnosis of mitochondrial diseases has limited correlation with phenotype. The same gene mutation can cause different phenotypes. Similarly, the same phenotype can be caused by many different gene mutations. So far, only a few TTC19 variants have been reported, and TTC19 gene mutations have been reported in 20 patients from 11 families^2^, ^7^, ^8^, ^9^, ^10^, ^11^, ^12^, ^13^, ^14^, ^15^, ^16^ (Table 1). All patients have homozygous mutations with large clinical phenotypic differences that are not easily attributed to specific syndromes.

**TABLE 1:** Comparison of clinical, biochemical, and neuroimaging in patients with TTC19 mutations.

Author	Gender	Origin	Age at onset	Signs/Symptoms at onset	Elevated lactate (Blood/CSF)	Evolution	Functional status at last contact	TTC19 mutations	Complex III deficit in muscle	Neurological status	MRI	MRS Lactate peak	Ref
Ghezzi	F	Italian	Late infancy	Learning disability and gait ataxia	Normal (plasma)	Slowly progressive	Wheelchair dependent	c.656T>G;p.Leu219*	19%	Ataxia Dysphagia Dysarthria Enhanced tendon reflexes	Leukoencephalopathy, hyperintense caudate nucleus, and cerebellar atrophy	+	2
	M	Italian	Late infancy	Learning disability and gait ataxia	NA	Rapidly progressive	Non‐ambulant		14%	Ataxia Dysphagia Dysarthria	NA	NA	
	F	Italian	Late infancy	Regression of language and gait ataxia	High	Progressive and rapid worsened by cardiorespiratory arrest	Non‐ambulant		17%	Ataxia Dysarthria Peripheral neuropathy	Cerebellar atrophy	NA	
	M	Italian	42 years	Weakness of all extremities	NA	Rapidly progressive	Deceased	c.517C>T;p.Gln173*	8%	Ataxia Dysarthria Peripheral neuropathy Spasticity	NA	+	
Nogueira	M	Portuguese	27 years	Mood disorder and gait ataxia	High	Rapidly progressive	Deceased	c.963_966del;p.Ala321Alafs*8	30%	Ataxia Dysphagia Dysarthria Enhanced tendon reflexes Peripheral neuropathy Spasticity	Olivo‐ponto‐cerebellar atrophy and hypersignal changes in caudate, putamen, cerebellar dentate nucleus, medial midbrain, and medullary olives on T_2_‐weighted sequences	−	16
	M	Portuguese	12 years	Compulsive lying	NA	Rapidly progressive	Deceased	NA	31%	Ataxia Dysphagia Dysarthria Enhanced tendon reflexes Spasticity		−	
	M	Portuguese	15 years	Aggressive behavior	NA	Rapidly progressive	Deceased	NA	33%	Ataxia Dysphagia Dysarthria Enhanced tendon reflexes Peripheral neuropathy Spasticity		−	
	F	Portuguese	34 years	Aggressive behavior	NA	Rapidly progressive	Deceased	c.963_966del;p.Ala321Alafs*8	39%	Ataxia Dysphagia Dysarthria Enhanced tendon reflexes		−	
Atwal	M	Hispanic	1 years	Developmental delay and language regression	NA	Slowly progressive	Ambulant	c.964_967del/c.577G4A; p.Trp186*/p.Gly322‐Metfs*8	I + III 79%, II 52%, II + III 36%, IV 46%	NA	Progressing T_2_ high signal lesions in putamen, caudate body, and brainstem	−	8
Morino	F	Japanese	31 years	Dysarthria	+/NA	Rapidly progressive	Wheelchair dependent	c.829C4T;p.Glu277*	NA	Ataxia Dysarthria Enhanced tendon reflexes pes cavus	Cerebellar atrophy and bilateral T_2_ high intensity at inferior olives	−	15
Ardissone	M	Moroccan	4 years	Cognitive impairment (language regression) ataxia	+	Rapidly progressive	Wheelchair dependent	c.782_786del;p.Glu261Glyfs*8	50%	Spasticity	Bilateral striatal necrosis	NA	7
Melchionda	F	Arab	18 months	Unsteady gait with frequent falls	–/NA	Rapidly progressive	Bed‐ridden	c.213_229dup;p.Gln77Argfs*30	28%	Ataxia Dysarthria Enhanced tendon reflexes	Mild cerebellar vermis atrophy and bilateral symmetrical T_2_ high intensity lesions in lentiform nucleus with cavitated aspects on FLAIR sequence	NA	13
Kunii	M	Japanese	25 years	Mood disorder and gait ataxia	−/−	Slowly progressive	Wheelchair dependent	c.157_158dup;p.Pro54Alafs*48	74.60%	Ataxia Dysarthria deep sensory impairment Enhanced tendon reflexes Peripheral neuropathy Spasticity pes cavus	Cerebellar atrophy and symmetric T_2_ high intensity lesions in the inferior olives and adjacent to periaqueductal gray matter	−	12
Mordaunt	F	Iraqi	8 years	Developmental delay	−/−	Rapidly progressive	Deceased	c.937C>T;p.Q313X	4%–6%	Ataxia Dysphagia Hypotonia	Mild cerebellar and cerebral volume loss, bilateral patchy high Signal T_2_/FLAIR, and hypo‐toiso‐intense T_1_ foci within the lentiform nuclei	NA	14
Koch	M	Turkish	Neonatal	Lactic acidosis	+/NA	Slowly progressive	Vegetative state	c.971T>C;p.Leu324Pro	28%	Ataxia Dysphagia Dysarthria Enhanced tendon reflexes Spasticity Hypotonia	Symmetrical T_2_‐weighted hyperintensities of basal ganglia and periventricular white matter	NA	11
	M	Austrian	19 months	Global developmental delay, ataxia, dysarthria, hypotonia	+/NA	Slowly progressive	Wheelchair dependent	c.656T>G;p.Leu219*d	31%	Ataxia Dysphagia Dysarthria Enhanced tendon reflexes Spasticity Hypotonia	Bilateral T_2_‐weighted hyperintensities of the putamen, the caudate nucleus, and the mesencephalon periaqueductal gray matter	NA	
	M	Romanian	3 years	Developmental delay, ataxia, regression, hypotonia	−/−	Slowly progressive	Wheelchair dependent	c.554T>C;p.Leu185Pro	Normal	Ataxia Dysphagia Dysarthria Enhanced tendon reflexes Spasticity Hypotonia	Hyperintensities of nucleus lenticularis and nucleus caudatus, loss of volume in the putamen, and cerebral atrophy	+	
	F	Romanian	6 years	Mild developmental delay, hypotonia	NA	Slowly progressive	Ambulant		NA	Enhanced tendon reflexes Hypotonia	Hyperintensities in caput caudate nucleus and basal parts of the putamen, increased interfoliar spaces in cerebellum	NA	
Conboy	M	Kuwaiti	3.5 years	Recurrent stroke‐like episodes, developmental delay	+	Slowly progressive	Wheelchair dependent	c.150_153de;p.Arg52fs	NA	Ataxia Dysphagia Dysarthria	Bilateral symmetrical T_2_‐weighted hyperintensities and cystic changes of putamina and the caudate nuclei	+	9
Habibzadeh	M	Iranian	7 years	Aggressive behavior and hyperactivity	+/NA	Slowly progressive	Wheelchair dependent	c.581delG;p.Arg194Asnfs*16	NA	Ataxia Dysphagia Dysarthria Enhanced tendon reflexes Spasticity	Bilateral hypersignal changes in caudate bodies and lentiform nuclei on T_2_ and FLAIR, cerebellar atrophy	NA	10
The present study	M	Chinese	9 years	Progressive movement disorders, limb weakness, suspicious ataxia and peripheral neuropathy	NA	Slowly progressive	Ambulant	c.719_732del;p.Leu240Serfs*17	NA	Ataxia Dysarthria Enhanced tendon reflexes peripheral neuropathy pes cavus	Multiple abnormal signal changes in bilateral basal ganglia and medulla on T1, T2 and Flair images.	NA	

Abbreviations: CSF, Cerebrospinal fluid; F, female; M, male; MRI, magnetic resonance imaging; MRS, magnetic resonance spectroscopy; NA, not available; Ref, reference.

CIII deficiency is mainly associated with a wide range of clinical features, including infantile‐onset cardiomyopathy, childhood‐onset myopathy, childhood or adolescent‐onset spinocerebellar ataxia and psychosis, adult‐onset rapidly progressive multisystem failure with encephalomyopathy, and adult‐onset paroxysmal spinocerebellar ataxia.^16^, ^17^, ^18^, ^19^ A skeletal muscle biopsy showed reduced activity of MRC CIII. Brain MRI showed white matter degeneration, caudate nucleus, putamen, and lower olive nucleus high signal, accompanied by cerebellar pons atrophy. Some patients had peripheral neuropathy. The disease phenotype is heterogeneous and may be associated with low incidence. More reports are needed to explore the pathological mechanism and pathogenesis of the disease. For patients with similar symptoms above who were highly suspected with mitochondrial disease, whole exon gene detection could be used to assist in the diagnosis.

Bottani et al. proved that the TTC19 gene located on chromosome 17p12 encodes a TTC19 (tetrapeptide repeat structure protein), which is embedded in the mitochondrial inner membrane.^20^ Severe neurological functional deficiency and MRC III defects can be caused by the loss of TTC19.^20^ TTC19 is involved in the removal of the N‐terminal proteolytic fragment of the Rieske protein, which is produced when incorporated into complex III.^20^ Therefore, TTC19 participates in the functioning of complex III. The animal model emphasized the importance of the TTC19 gene. TTC19‐deficient mice showed slow neurological deficits and a lack of CIII2 activity in the liver and skeletal muscle.^21^ Similarly, Gheezi et al. showed that TTC19 deficiency resulted in a shortened lifespan, reduced fertility, adult movement disorders, and visual and motor dysfunction in adult Drosophila.^2^ The homozygous mutation found in our study caused the reading frame to change, resulting in the truncation of proteins containing 17 error‐coding amino acids. The lack of expression of biologically functional proteins leads to progressive ataxia, dysarthria, basal ganglia lesions, and peripheral neuropathy in our patient. However, we did not obtain muscle biopsies to confirm the functional inhibition of complex III in muscle fibroblasts.

Symptoms of the TTC19 mutation are often manifested as speech or cognitive functional degradation in young patients, while they are characterized by mental disorders in adults. A 25‐year‐old Japanese patient had a mood disorder.^12^ A 27‐year‐old Portuguese patient had mood disorders and gait ataxia.^16^ A 1‐year‐old Hispanic patient had developmental delays and language regression.^8^ Ardissone reported a 4‐year‐old patient who suffered from language regression.^7^ Our patient showed limb weakness, weird posture, and involuntary movement. He had normal memory and executive function. However, his academic performance at school was poor. A psychiatrist suspected that he suffered from an attention deficit, hyperactivity disorder, mild mental retardation, and exhibitionism.

The boy showed involuntary movement, which was not mentioned in previous literature reports with TTC19 mutations. The pathogenesis of involuntary movement may be speculated to the damage to striatal neurons. The decrease of γ‐aminobutyric acid (GABA) transmitted from the striatum to the lateral thalamus leads to the obstruction of the striatum cortical pathway, which subsequently brings on the disinhibition of the thalamus.^22^

The patient had symptoms such as progressive movement disorders, limb weakness, and suspicious ataxia, which were consistent with the clinical characterizations of TTC19 gene mutations. However, the patient had involuntary movement and cervical cord lesions, which had not been reported in the previous literature, which was regarded as expanding the disease phenotype. Cervical cord lesions could be paid attention to in patients with TTC19 gene mutations.

## 5 FUNDING INFORMATION

Yunnan Health Training Project of High Level Talents (H‐2017032), The Major Science and Technology Special Project of Yunnan Province (202102AA100061), National Natural Science Foundation of China (82160233), Yunnan Basic Research Funding (202201AY070001‐071), Reserve Talents Project of Young and Middle‐aged Academic and Technical Leaders in Yunnan Province (202305AC160045).

## 6 AUTHOR CONTRIBUTIONS

XX and JR designed the study. LL and CW performed the genetic analysis and bioinformatics evaluations. LL and XL drafted the manuscript. XL conducted the clinical evaluations. All authors analyzed the data and approved the final manuscript.

## 7 CONFLICT OF INTEREST STATEMENT

The authors declare no conflicts of interest.

8

## 9 INFORMED CONSENT

All included patients or their deputies provided written informed consent.

## Supporting information


Video S1

## Data Availability

The data that support the findings of this study are available from the corresponding author upon reasonable request.
